# Epidemiological features of seasonal influenza transmission among 11 climate zones in Chinese Mainland

**DOI:** 10.1186/s40249-024-01173-9

**Published:** 2024-01-10

**Authors:** Xiaohan Si, Liping Wang, Kerrie Mengersen, Wenbiao Hu

**Affiliations:** 1 Ecosystem Change and Population Health Research Group, School of Public Health and Social Work, Queensland University of Technology, Brisbane, QLD 4059 Australia; 2 Information Center, Chinese Center for Disease Control and Prevention, Beijing, 102206 China; 3 School of Mathematical Sciences, Queensland University of Technology, Brisbane, QLD 4000 Australia

**Keywords:** Seasonal influenza, Köppen Geiger climate zones classification system, Chinese Mainland, Seasonality decomposition, Local indicators of spatial association

## Abstract

**Background:**

Previous studies provided some evidence of meteorological factors influence seasonal influenza transmission patterns varying across regions and latitudes. However, research on seasonal influenza activities based on climate zones are still in lack. This study aims to utilize the ecological-based Köppen Geiger climate zones classification system to compare the spatial and temporal epidemiological characteristics of seasonal influenza in Chinese Mainland and assess the feasibility of developing an early warning system.

**Methods:**

Weekly influenza cases number from 2014 to 2019 at the county and city level were sourced from China National Notifiable Infectious Disease Report Information System. Epidemic temporal indices, time series seasonality decomposition, spatial modelling theories including Moran’s *I* and local indicators of spatial association were applied to identify the spatial and temporal patterns of influenza transmission.

**Results:**

All climate zones had peaks in Winter-Spring season. Arid, desert, cold (BWk) showed up the first peak. Only Tropical, savannah (Aw) and Temperate, dry winter with hot summer (Cwa) zones had unique summer peak. Temperate, no dry season and hot summer (Cfa) zone had highest average incidence rate (IR) at 1.047/100,000. The Global Moran’s *I* showed that average IR had significant clustered trend (*z* = 53.69, *P* < 0.001), with local Moran’s *I* identified high-high cluster in Cfa and Cwa. IR differed among three age groups between climate zones (0–14 years old: *F* = 26.80, *P* < 0.001; 15–64 years old: *F* = 25.04, *P* < 0.001; Above 65 years old: *F* = 5.27, *P* < 0.001). Age group 0–14 years had highest average IR in Cwa and Cfa (IR = 6.23 and 6.21) with unique dual peaks in winter and spring season showed by seasonality decomposition.

**Conclusions:**

Seasonal influenza exhibited distinct spatial and temporal patterns in different climate zones. Seasonal influenza primarily emerged in BWk, subsequently in Cfa and Cwa. Cfa, Cwa and BSk pose high risk for seasonal influenza epidemics. The research finds will provide scientific evidence for developing seasonal influenza early warning system based on climate zones.

**Supplementary Information:**

The online version contains supplementary material available at 10.1186/s40249-024-01173-9.

## Background

Seasonal influenza has long been recognised as a human infectious disease of major importance causing heavy disease burden, with globally estimated deaths of 35 to 60 million due to its high transmission rate and pathogenicity [1]. Since the establishment of National Influenza Center in 1957 and the national surveillance network in 2000, the seasonal influenza was monitored as a notifiable infectious disease in Chinese Mainland via both clinically and laboratory confirmation [2].

Previous studies showed that temperature and humidity were found strongly related to seasonal influenza transmissibility but brought about varying changes in the features of influenza transmission [3–5]. Low temperature and low humidity were favoured by the influenza virus in temperate regions, whereas influenza epidemics prefer warm temperature and high humidity in tropical and subtropical zones [4].However, most previous ecological studies in Chinese Mainland selected study sites on the city or provincial level but rarely on nationwide, neglecting the possibility of changes in the epidemiological characteristics of seasonal influenza due to ecosystem diversity within a single administrative area [6, 7].

One of our previous preliminary studies in Gansu Province, China observed significant differences of epidemic features among climate zones exists [7]. But this study only involved climate zones in a province, the difference among other climate zones in Chinese Mainland remain unclear. Moreover, few studies have systematically analysed spatial and temporal patterns at various climate zones and explored potential correlations among different climate zones nationwide. A general summary and conclusion of summarizing seasonal influenza spatial and temporal based on regions sharing similar climatic features were not conducted.

It is reasonable to propose that the classification of climatic zones throughout Chinese Mainland will allow for a better presentation of regions with similar epidemiological characteristics, and thus a more obvious demonstration of the epidemiological characteristics of seasonal influenza from a spatial and temporal perspective, contributing to influenza early warning. The Köppen Geiger classification, a climate zones classification system using ecosystem indicators as classification criteria, rather than the classical criteria of only latitude and longitude (tropical, subtropical and temperate zones), provides us with a practical way to test such proposal [8].

This study aims to compare the spatial and temporal epidemiological characteristics of seasonal influenza among climatic zones in Chinese Mainland, and to identify the feasibility of linking regional ecological conditions with local seasonal influenza activities.

## Methods

### Data collection and study settings

#### Study site and climate zone classification

We choose Chinese Mainland as our study site where had enormous population size with over 1.4 billion people at the end of 2019, covering a territory of approximately 9.6 million square kilometres (including inner land and territorial water) and crossing over 50 degrees longitude as well as multiple climate zones.

We used the Köppen Geiger climate classification system which was promoted by Wladimir Köppen and Rudolf Geiger in late 19 centuries [9]. In this study, a more accurate updated Köppen Geiger climate map which was drawn based on meteorological observations data until 2016 was used, comparing the map used in our preliminary study [7, 8]. Köppen Geiger climate classification assigned 31 climate zones globally. The classification is based on 5 main climates with seasonal patterns and threshold values of monthly air temperature and precipitation. The 5 main climates are A (Tropical), B (Arid), C (Temperate), D (Continental) and E (Polar). The subtypes of climate under these 5 main climates are based on historical average regional precipitations and temperature [8, 10].

**Fig. 1:**
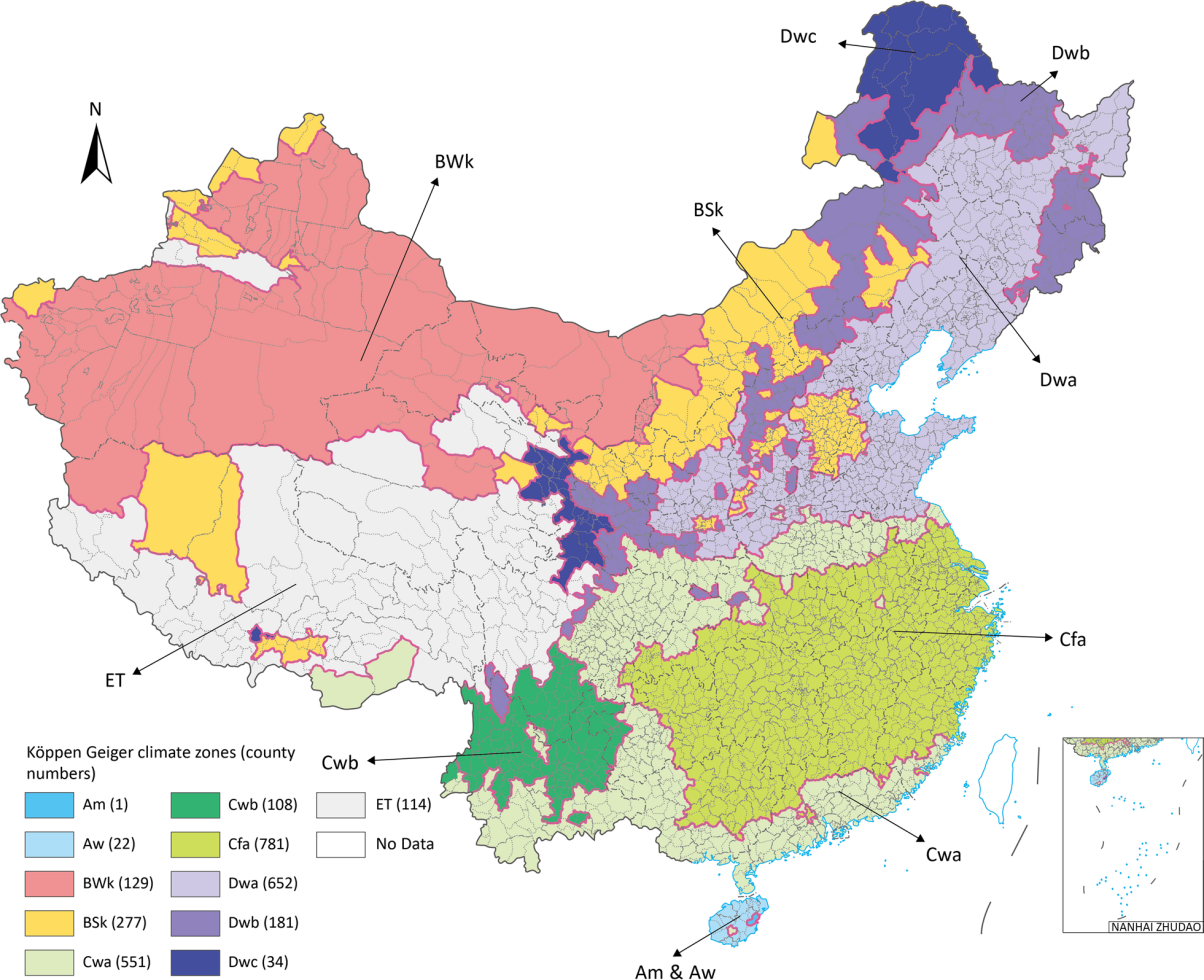
Köppen Geiger climate zones distribution in Chinese Mainland with inner county numbers. Map approval No.: GS (2023) 4611

In China, totally 11 out of 31 Köppen Geiger climate zones were identified, which are Am (Tropical, monsoon), Aw (Tropical, savannah), BWk (Arid, desert, cold), BSk (Arid, steppe, cold), Cwa (Temperate, dry winter, hot summer), Cwb (Temperate, dry winter, warm summer), Cfa (Temperate, no dry season, hot summer), Dwa (Continental, dry winter, hot summer), Dwb (Continental, dry winter, warm summer), Dwc (Continental, dry winter, cold summer) and ET (Polar, tundra). On county level, we assigned each county to a single climate zone (Fig. 1). For counties across multiple climate zones, we marked it with the climate zones code which takes the largest area in this county. The Köppen Geiger climate zones distribution on county level is shown as in Fig. 1.

The geographical data of Köppen Geiger climate classification file at 1 km resolution updated by Beck in 2016 was obtained from internet [8]. The Chinese Mainland map was collected from Ministry of Natural Resources of China (http://bzdt.ch.mnr.gov.cn).

#### Influenza surveillance and notification data

In Chinese Mainland, all cases are reported to Notifiable Infectious Diseases Reporting Information System (NIDRIS) by more than 70 thousands medical institutions involving all townships, communities and counties or above levels, which has been widely used in the previous studies [11, 12]. All data used in this study was collected from NIDRIS, including both clinically confirmed and laboratory-confirmed notified cases on county level. The weekly incidence rate was calculated at per person week, via weekly reported cases number divided by the average yearly local population during our study period. The influenza cases were clinically diagnosed or laboratory confirmed following the criteria and procedure in Chinese Center for Disease Control and Prevention Influenza sentinel surveillance protocol and technical guidelines.

Weekly influenza cases notification numbers confirmed by both clinical and laboratory were collected at county level from NIDRIS from Week 40, 2014 to Week 39, 2019, including total 5 surveillance years. A surveillance year was defined as from week 40 in a year to week 39 in next year. Age group specific cases notification number was collected on city level from sentinel hospitals in NIDRIS from Week 40, 2015 to Week 39, 2019 in total 4 surveillance years. Three age groups were classified in this study: 0–14 years old, 15–64 years old and above 65 years old. The annual population size data at county level from 2014 to 2019 was obtained from China National Bureau of Statistics (http://www.stats.gov.cn).

### Statistical analysis

#### Epidemic temporal indices analysis by climate zone

The cases number we collected was assigned to climate zones by county. The original counties notifications number records were archived by unique administrative code in each county.

In our previous study on influenza transmission features in climate zones, three temporal indices of Frequency index (α), Duration index (β) and Intensity index (γ), showed good performance on characterizing disease temporal patterns and their changes [13]. In this study, these temporal indices were also used to evaluate and compare the changes of seasonal influenza epidemic features between climate zones from temporal perspective.

The Frequency index (α) is defined as:

$$a = CW/TW,$$

where CW is the cumulative number of weeks in which one or more cases were reported, TW is the total number of weeks over the study period (261 weeks). The risk of influenza cases occurring within this study period increases as the index closes to 1.

The Duration index (β) was defined as:

$$\beta = CW/PV,$$

where CW is described above, and PV is the total number of prevalent waves during the entire study period. PV started from cases that continuously appeared to when no cases occurred. This index indicates the epidemic wave numbers during the study periods. It was used as an indicator of the effectiveness of the disease control and prevention measures by public health administrative apartments. For example, if local public health authorities can respond and implement disease control measures fast, the duration of the epidemic will be short and drop to no new cases quickly with low duration index. Otherwise, the epidemic wave will be hard to disappear, shown as the large value of duration index [13].

The Intensity index (γ) can be described as the magnitude of influenza activity in a prevalent wave. It is defined as:

$$\gamma = IR/PV,$$

where IR is the incidence rate (per 100,000 people) during 2014–2019, and PV is described above.

The intensity index (γ) focuses on the intensity of the disease epidemic during weeks of consecutive cases. The smaller the number of epidemic waves, the greater the intensity index (γ), which means that more cases concentrated in this interval.

One-way Analysis of Variance (ANOVA) was used to test if there are significant differences of weekly average cases number and incidence rates exists between different climate zones [14].

#### Seasonality decomposition and cross-correlation analysis

The classic time series decomposition was applied to separate seasonal factor from the original incidence rate time series data in each age groups and climate zones [15]. Seasonal factor was then used for the cross-correlation analysis to find lag effect among climate zones [16]. Time series decomposition and cross-correlation analysis was conducted in R (version: 4.2.2; R Foundation for Statistical Computing).

#### Spatial cluster analysis

The method of local indicators of spatial association (LISA) was adopted in our study, using local Moran's *I* measure to quantify spatial autocorrelation of the county and city level incidence rate throughout the whole nation [17]. The Kriging Method is usually used to generate predictions of neighbor regions of known data area based on the spatial distances and its changing patterns [18]. In this study, the Kriging Method was used to predict and conclude the potential high average incidence rate area and spatial cluster patterns via heat map.

Moran's *I* had value within [− 1, 1] interval, where the value approached to 1 when the variable tends to be clustering with similar values and to − 1 if variables are unrelated [19]. Moreover, Moran's *I* = 0 means no spatial autocorrelation exists. The high value areas can be identified as hot spots (high–high cluster) if the areas have a high value neighborhood and turned to low-low cluster if neighboring value is low. Furthermore, we identified the climate zones where hot spots located in to discover the high incidence rate cluster area. All spatial analyses were conducted via ArcGIS Pro (version: 3.0.0; Esri Inc., Redlands, CA, USA) and R (version: 4.2.2; R Foundation for Statistical Computing).

## Results

Our results find that seasonal influenza had yearly circulation and its incidence rates exhibited distinct spatial and temporal patterns in different Köppen Geiger climate zones. Cfa had the highest epidemic intensity while the BWk and Cwb zones had most prevalent waves. High risk clusters were primarily located in the southeast and southern coastal areas of Chinese Mainland, especially in Cfa and Cwa.

### Descriptive analysis

As Table 1 showed, the climate zones in Chinese Mainland included in this study are Am, Aw, BWk, BSk, Cwa, Cwb, Cfa, Dwa, Dwb, Dwc and ET. The mean weekly incidence rate varied among these climate zones, with the highest incidence rate in AW at 1.654 per 100,000 people and the lowest in BWk at 0.254 per 100,000 people. The peak mean incidence rate also varied, with the highest rate in BSk at 2938.8 per 100,000 people and the lowest in AW at 4.625 per 100,000 people. The mean weekly cases number and peak mean cases number were highest in Cwa, with 4502.427 and 18,580 respectively. The population proportion was the highest in Cfa at 31.623%, and the counties number was the highest in Cwa at 551. The high values were found in the Cwa on both average incidence rate and reported cases number. Cfa had the highest average and peak cases number with largest population of 31.623% in Chinese Mainland.

**Table 1:** Descriptive analysis on climate zones

Climate zones	Mean weekly incidence rate (per 100,000 people)	Peak mean incidence rate (per 100,000 people)	Mean weekly cases number	Peak mean cases number	2019 Population proportion (%)	Counties number	Area (km^2^)	Area proportion (%)
Am	1.189	13.123	10.951	60.8	–	1	–	–
Aw	1.654	4.625	74.007	266	0.553	22	37,299	0.41
BWk	0.254	1.519	98.527	402.6	2.147	129	2,069,295	22.54
BSk	0.678	3.172	759.069	2938.8	8.327	277	912,696	9.94
Cwa	1.053	5.601	4502.427	18,580	25.345	551	1,024,624	11.16
Cwb	0.319	1.215	102.227	395.6	3.354	108	308,582	3.36
Cfa	1.407	9.385	5381.338	23,260.8	31.623	781	1,245,406	13.57
Dwa	0.675	3.701	2888.031	14,368.8	24.630	652	992,893	10.82
Dwb	0.455	2.043	182.285	577.6	3.068	181	683,447	7.45
Dwc	0.427	2.980	17.888	81.6	0.427	34	310,456	3.38
ET	0.762	5.212	8.4	46.6	0.522	114	1,594,138	17.37

Am: Tropical, monsoon, Aw: Tropical, savannah, BWk: Arid, desert, cold, BSk: Arid, steppe, cold, Cwa: Temperate, dry winter, hot summer, Cwb: Temperate, dry winter, warm summer, Cfa: Temperate, no dry season, hot summer, Dwa: Continental, dry winter, hot summer, Dwb: Continental, dry winter, warm summer, Dwc: Continental, dry winter, cold summer, ET: Polar, tundra, –: Not applicable

Additional file 1: Table S1 presents the incidence rates of different age groups within climate zones. Cwa had the highest maximum incidence rates across all age groups. Notably, the age group 0–14 years generally experiences the highest maximum incidence rate in most climate zones. In terms of average incidence rates, Cwa maintains the highest values across all age groups. For age over 65, BSk had the highest average incidence rate and Cwa also remain at high incident level. Additional file 1: Fig. S1 showed the incidence rate in each age group, which has similar conclusion as above.

### Temporal epidemic indices

From Table 2 we can see the Cfa zone had the highest average incidence rate of 1.3 per 100,000 people and intensity index of 0.14. The BSk zone had the highest duration index of 29.11, and the Dwb zone had the highest frequency index of 0.08. The BWk and Cwb zones had most 15 prevalent waves, while the ET zone had the fewest waves, with only four prevalent waves. Except ET and Am, all included counties in other climate zones had positive notification every week in all 5 surveillances years. All the lowest temporal epidemic indices were found in ET. Cwb had same frequency index as BWk which remain low. The duration index remains at a high level in most climate zones, indicating the yearly round circulated features of seasonal influenza.

**Table 2:** Temporal epidemic indices summary table

Climate zone	Cumulative weeks (CW)	Total study weeks (TW)	Prevalent waves (PV)	Average incidence rate (IR)	Duration Index	Frequency Index	Intensity Index
Am	115	262	6	0.43	0.44	19.17	0.07
Aw	262	262	12	0.53	1.00	21.83	0.04
BWk	262	262	15	0.24	1.00	17.47	0.02
BSk	262	262	9	0.60	1.00	29.11	0.07
Cwa	262	262	9	0.87	1.00	29.11	0.10
Cwb	262	262	15	0.25	1.00	17.47	0.02
Cfa	262	262	9	1.30	1.00	29.11	0.14
Dwa	262	262	7	0.60	1.00	37.43	0.09
Dwb	262	262	5	0.39	1.00	52.40	0.08
Dwc	258	262	10	0.40	0.98	25.80	0.04
ET	239	262	4	0.45	0.91	59.75	0.11

Am: Tropical, monsoon, Aw: Tropical, savannah, BWk: Arid, desert, cold, BSk: Arid, steppe, cold, Cwa: Temperate, dry winter, hot summer, Cwb: Temperate, dry winter, warm summer, Cfa: Temperate, no dry season, hot summer, Dwa: Continental, dry winter, hot summer, Dwb: Continental, dry winter, warm summer, Dwc: Continental, dry winter, cold summer, ET: Polar, tundra

We also performed One-Way ANOVA on both average weekly cases number (*F* = 68.96, *P* < 0.001) and incidence rate (*F* = 6.596, *P* < 0.001). The ANOVA test results indicated the cases number and incidence rate had significant differences between climate zones in this study. Each age group incidence rate also had significant differences between climate zones in ANOVA (0–14 years old: *F* = 26.80, *P* < 0.001; 15–64 years old: *F* = 25.04, *P* < 0.001; Above 65 years old: *F* = 5.27, *P* < 0.001).

### Seasonal patterns analysis

From Table 3, totally three kinds of seasonal epidemic peaks were identified, which are primary and secondary peaks in winter-spring season and summer season.

**Table 3:** The seasonal peaks of incidence rate in each climate zones with appearance rank order in Chinese Mainland, 2014–2019

	Winter-spring peaks		Summer peaks
	First peak weeks (rank)	Second peak weeks (rank)	
Am	5 (4)	11 (3)	–
Aw	4 (3)	7 (1)	–
BSk	4 (3)	13 (5)	–
BWk	50 (1)	12 (4)	–
Cfa	4 (3)	10 (2)	26
Cwa	4 (3)	13 (5)	29
Cwb	4 (3)	13 (5)	–
Dwa	4 (3)	13 (5)	–
Dwb	4 (3)	12 (4)	–
Dwc	52 (2)	13 (5)	–
ET	4 (3)	7 (1)	–

Am: Tropical, monsoon, Aw: Tropical, savannah, BWk: Arid, desert, cold, BSk: Arid, steppe, cold, Cwa: Temperate, dry winter, hot summer, Cwb: Temperate, dry winter, warm summer, Cfa: Temperate, no dry season, hot summer, Dwa: Continental, dry winter, hot summer, Dwb: Continental, dry winter, warm summer, Dwc: Continental, dry winter, cold summer, ET: Polar, tundra, –: Not applicable

In winter-spring season, BWk and Dwc are the first two climate zones reach the primary peak on week 50 and week 52 correspondingly, then in other climate zones all reach the primary peak on week 4 and 5. Only Cwa had a unique peak in summer season on week 31. The second wave in spring season showed more significant differences. ET and Aw reached the peak on week 7, Cfa reached on week 10 and other zones on week 12–13. We also noticed in summer season, only Cwa had a significant epidemic wave and reach the peak on week 29, and a smooth peak on low level on week 30 in Cfa between summer and autumn. No summer season waves were observed in other climate zones. Cfa, Cwa, Cwb and Aw had earlier secondary peak in winter-spring epidemic than Dwa, Dwb and Dwc. The primary peak usually had higher transmission intensity than the secondary peak in winter-spring season.

The highest seasonal incidence rates are observed in season Cfa zone, with a value of 1.93, and in Aw zone, with a value of 1.07. On the other hand, the lowest seasonal incidence rates are observed in season 7 for all climate zones, with values ranging from 0.209 to 0.927. Overall, there seems to be a higher incidence rate during the winter season for most of the climate zones.

Additional file 1: Fig. S2 showed the seasonality decomposition results by age groups the seasonal factor in each climate zone. 0–14 years age group had dual peaks of different transmission intensity in winter-spring season which were not observed in other age groups. Also 0–14 age groups had early peak in winter. The peak time in climate zones in the same age group had no significant differences.

**Fig. 2:**
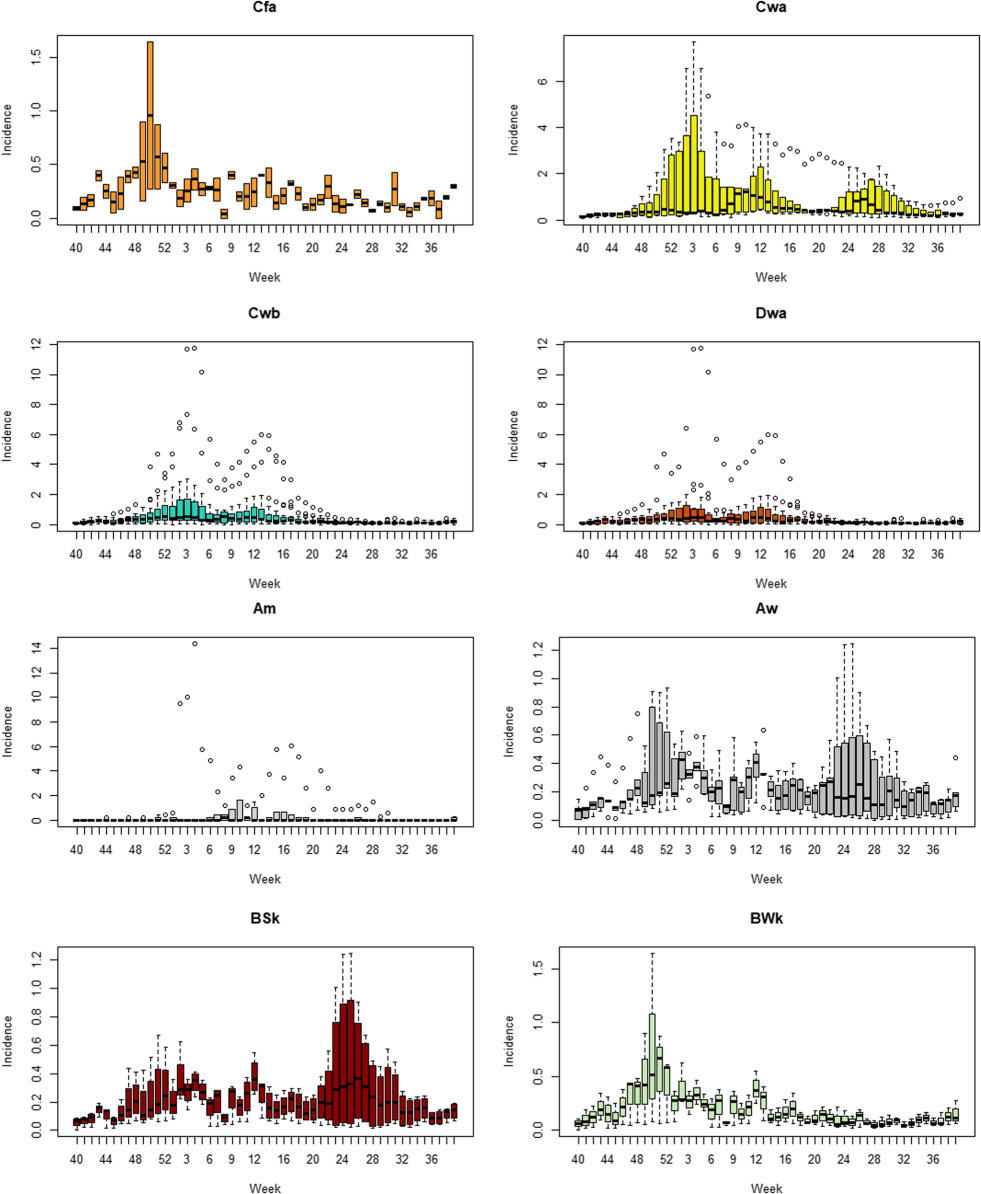

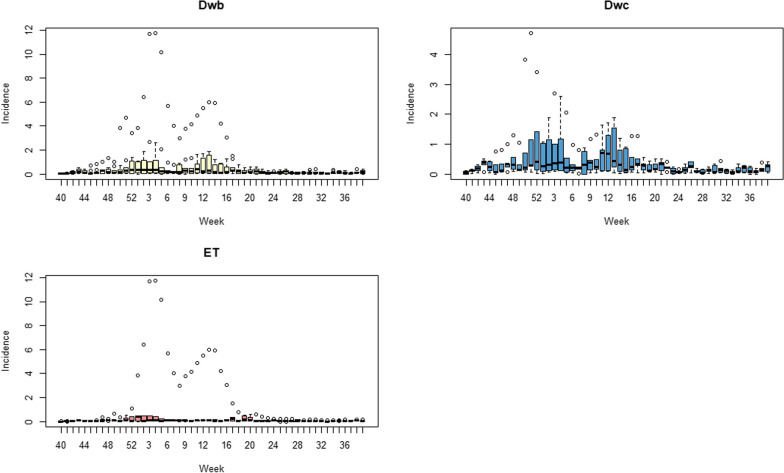
Boxplots of weekly influenza incidence rate by climate zone in Chinese Mainland 2014 to 2019

From the boxplot of average weekly incidence rate in Fig. 2, we can see all climate zones had dual peak in winter-spring season and only Cwa and Aw had the peak in summer season.

### Cross-correlation of seasonal factors among climate zones

The cross-correlation analysis of influenza activities seasonal factor in each climate zones showed significant lag effect exists between BWk and other climate zones (Additional file 1: Fig. S3). Dwc (northern China) had the smallest lag interval with BWk while Cfa (southern China) had the longest period (Additional file 1: Fig. S3).

### Spatial features of seasonal influenza in Chinese Mainland

#### Average incidence rate from 2014 to 2019 spatial clustering

**Fig. 3:**
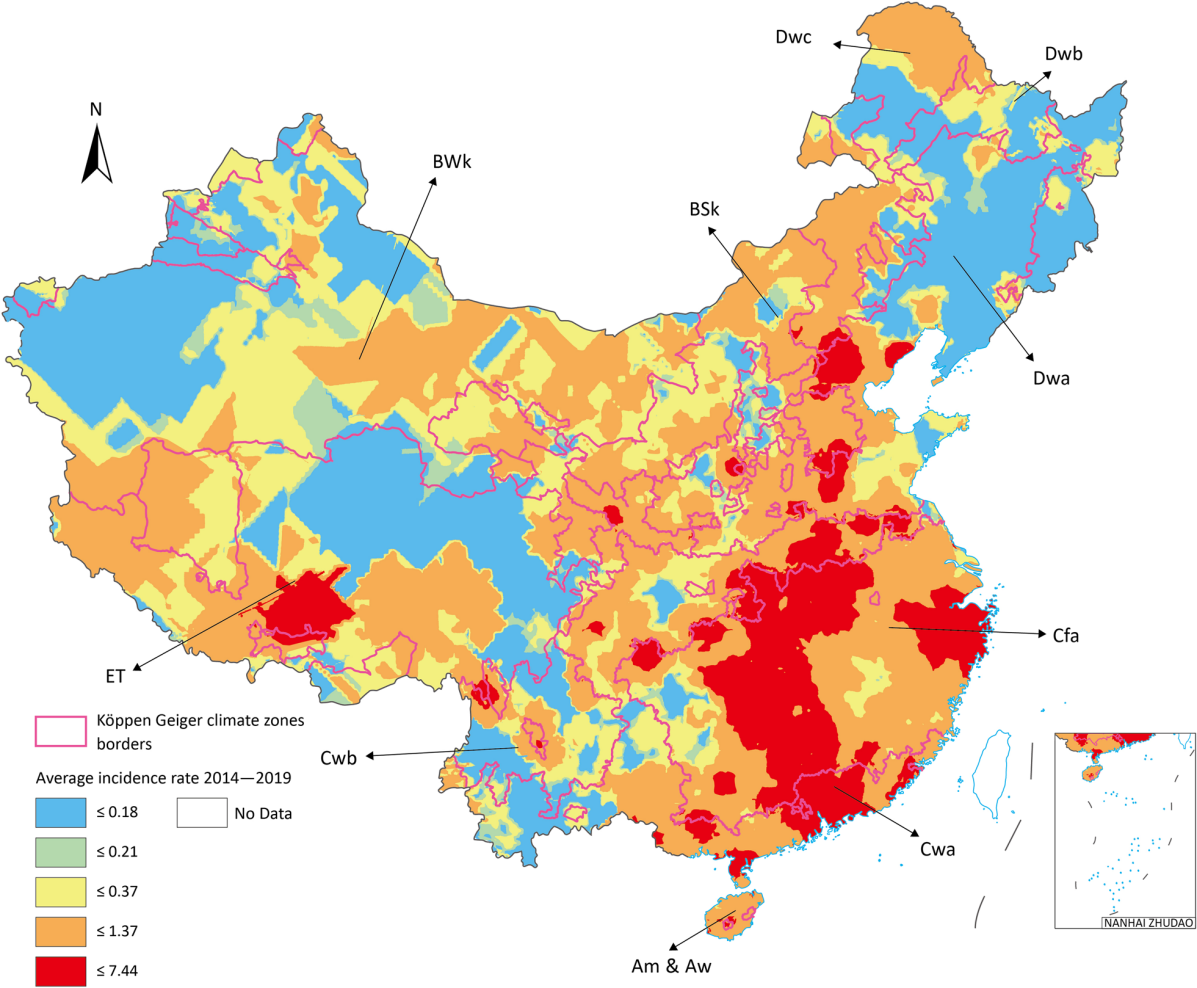
Heat map of average weekly incidence rate in Chinese Mainland 2014 to 2019. Map approval No.: GS (2023) 4611

We clearly see the high average incidence rate clustering in Cfa and Cwa, which are the two climate zones containing most proportion of population in Chinese Mainland. The heat map by Kriging Methods of average incidence rate showed the clear cluster of high-rate area in Cfa and Cwa (Fig. 3).

#### Local indicators of spatial association (LISA) of incidence rate in climate zones

**Fig. 4:**
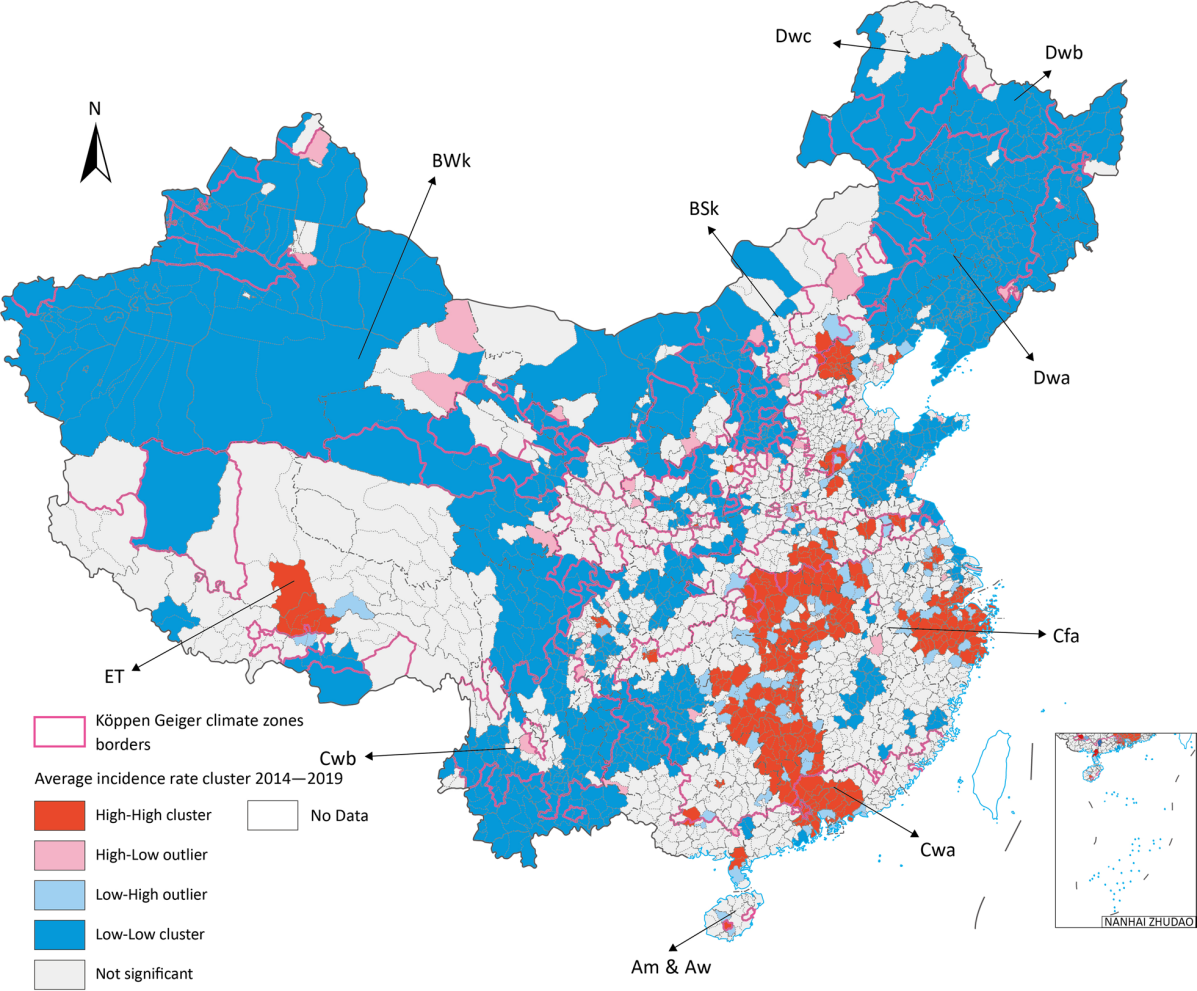
Average weekly incidence rate spatial clustering in Chinese Mainland 2014 to 2019. Map approval No.: GS (2023) 4611

From Fig. 4 of the hot clusters map, most high-high incidence rate cluster located in Cfa and Cwa. The Global Moran’s *I* showed that average incidence rate had significant clustered trend in Chinese Mainland (*z* = 53.69, *P* < 0.001). The local Moran’s *I* identified high-high incidence rate clusters in Cfa and Cwa. Besides two small area cluster point in Dwa and ET in very small certain range of area, which are Beijing and Lhasa, the China capital and province capital of Xizang correspondingly, where with the high population density. The low–low cluster mostly located in BWk and Dwa. Besides, high–low showed up in Dwb and low–high showed in Cfa.

**Fig. 5:**
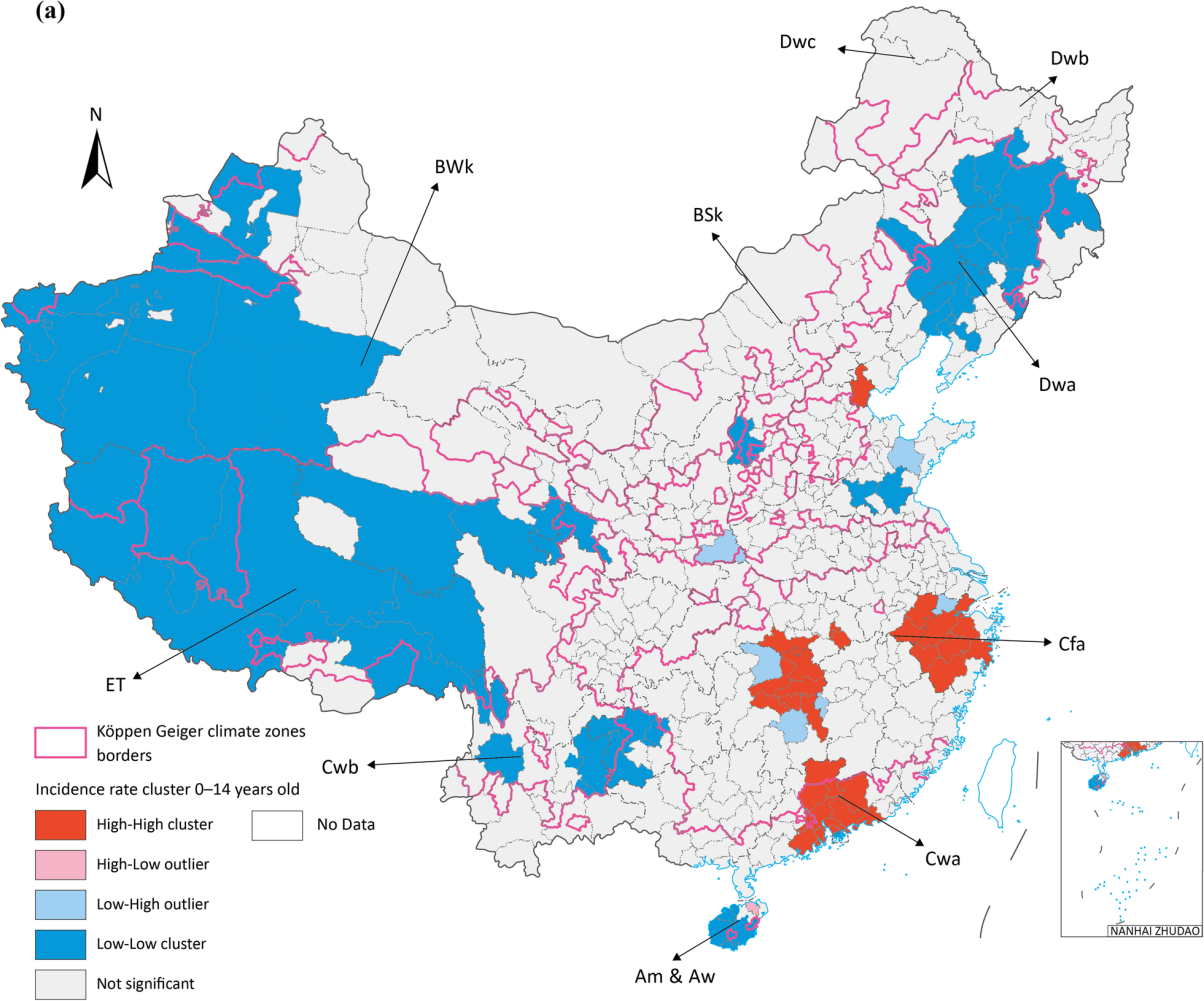

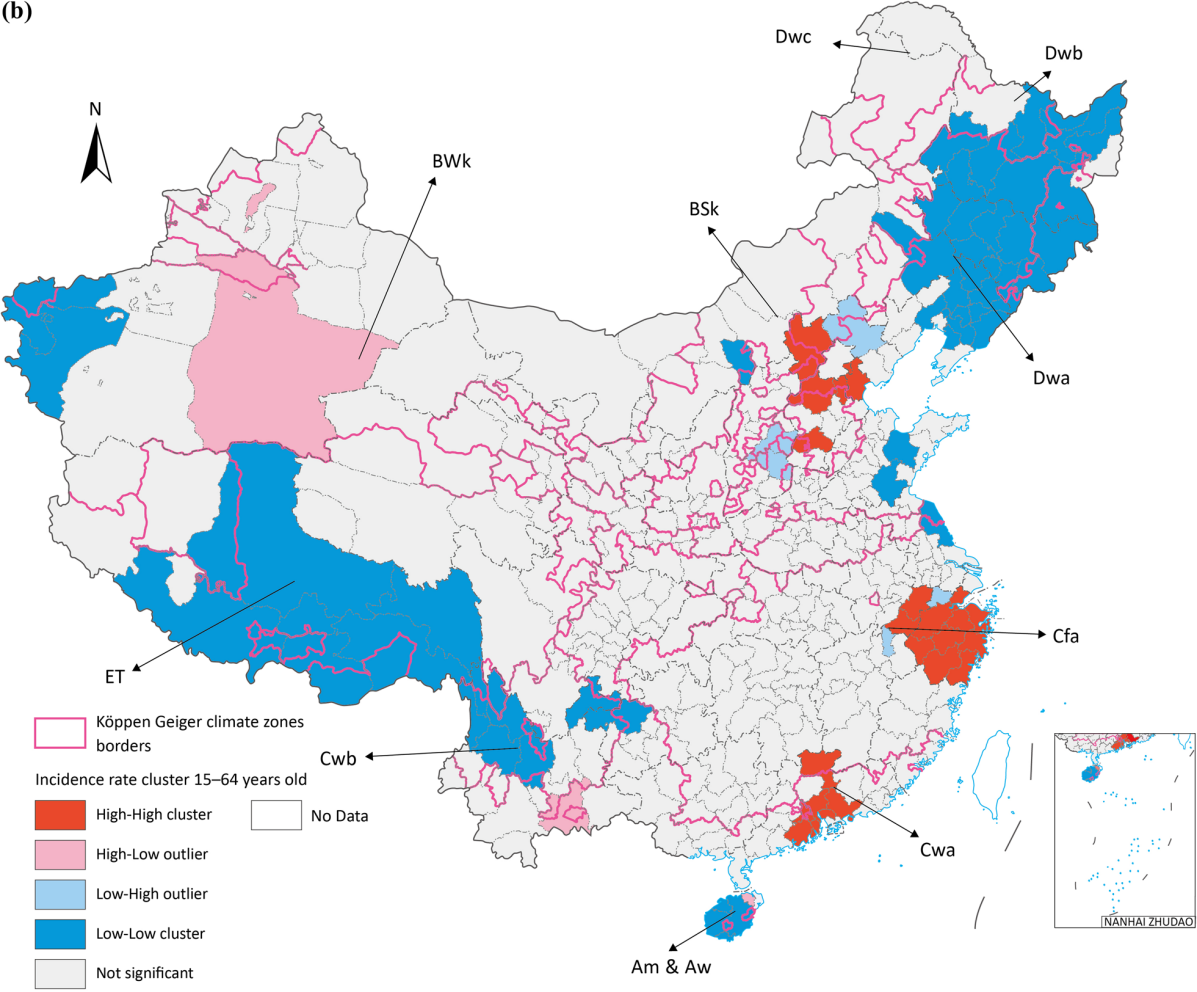

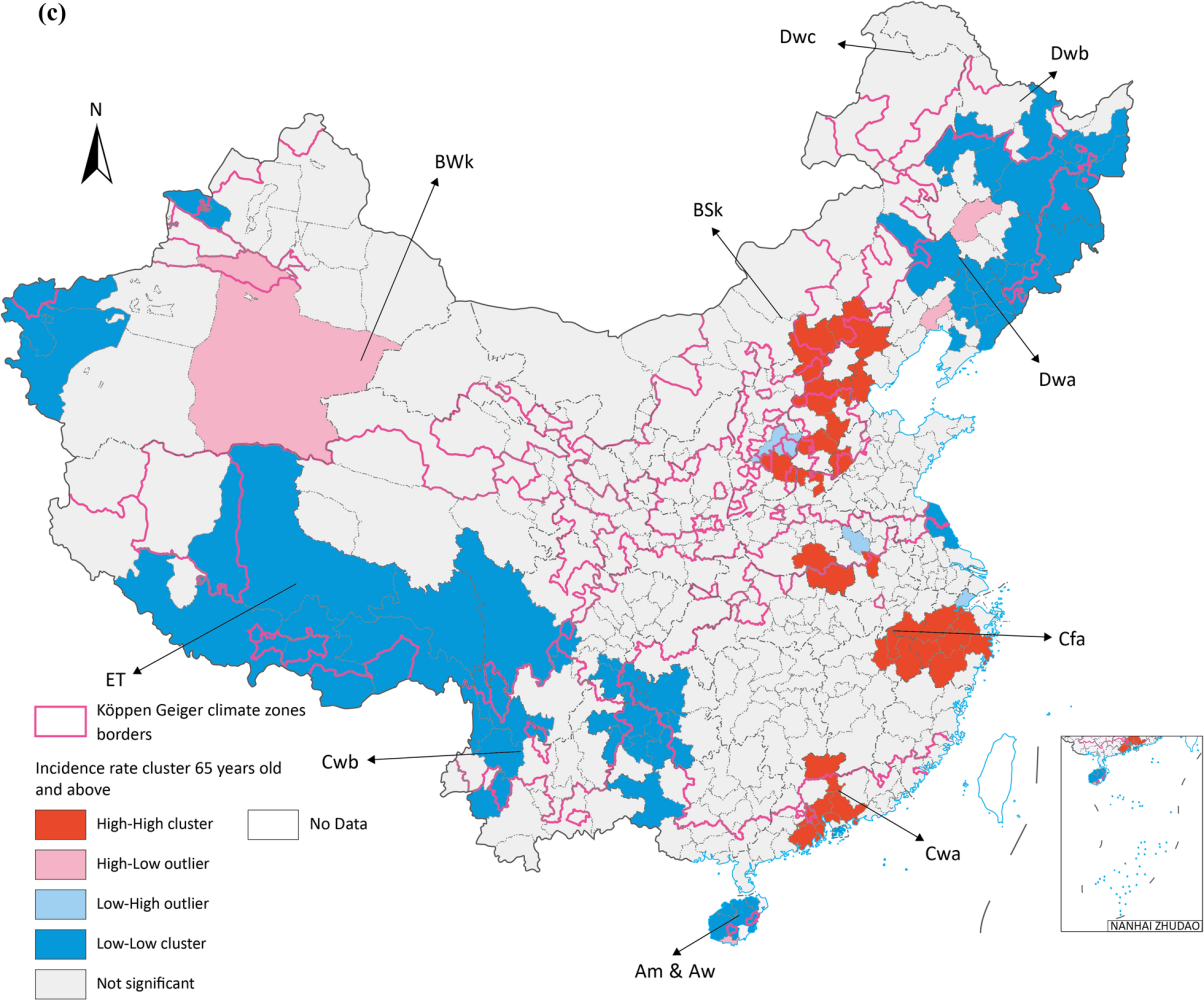
Average incidence rate by age groups spatial cluster in 2015 to 2019 (**a**) 0–14 years old (**b**) 15–64 years old (**c**) above 65 years old. Map approval No.: GS (2023) 4611

Two major clustering regions of high incidence rate cluster were identified in Cfa and Cwa, one in ET and the other one in Dwa, which are the potential prior incident area of seasonal influenza in Chinese Mainland (Fig. 4). All age groups had high-high clusters in Cfa and Cwa, especially for 0–14 groups all three major clusters are in these zones. BSk had both 15–64 and over 65 groups high risk clusters (Fig. 5).

## Discussion

This study utilized the Köppen Geiger classification system to investigate the variability of seasonal influenza epidemiological factors across different climate zones in Chinese Mainland based on county level influenza notification surveillance data. The findings indicated that seasonal influenza incidence rates exhibited distinct spatial and temporal patterns in ecological-based climate zones. High risk clusters were observed in Cfa and Cwa zones, primarily located in the southeast and southern coastal areas of Chinese Mainland. The Aw tropical area also had a high incidence rate, while the BWk zone in the north-western area showed a very low incidence rate. With the exception of Aw and Cwa zones, which exhibited an extra single peak in the summer season, all other climate zones experienced only two peaks during a continuous epidemic period in the winter-spring season. These spatial and temporal epidemic features align with previous studies in administrative northern China provinces/autonomous regions/municipalities [7, 20].

From a temporal perspective, the epidemic first appeared in the BWk and Dwc zones in the northwest and northeast regions of Chinese Mainland, characterized by cold and dry climates. Subsequently, the epidemic peak shifted southward to warmer and more humid climate zones. The longest lag interval with BWk of peak seasonal factor was observed in Cfa (southern China) and shortest in Dwc (northern China), supported our findings (Additional file 1: Fig. S3). One explanation for the two phases of different transmission intensity in all climate zones in winter-spring season is that the winter vacation of schools (usually in Week 2–6) and Chinese New Year public holiday (usually in Week 4–6) lead to a remission of the epidemic. Then the new semester usually started after the winter vacation increased the risk of influenza virus exposure and cross-infections and caused the increase of incidence rate again [21, 22]. Such peaks were only observed in the school-age population (0–14 years old) but not in other age groups is corroborating our opinion.

A previous study in nationwide on seasonal influenza promoted the summer influenza season in south China was initiated in Lingnan area, where covered Guangdong, Guangxi and Hainan Province, corresponding to Cwa and Aw climate zones in our study, and might playing the important role on maintaining the year round circulation of influenza [23]. The surveillance results in Guizhou Province, majorly located in Cwa and Cwb, showed dual peak in winter season and spring–summer season [24]. Zhejiang Province, in Cfa, had main peak from December to January of the next year followed by a peak in summer [25]. These studies evidenced our conclusion that Cwa, Cfa and Aw shared very similar temporal features of seasonal influenza and epidemiological analyses based on climate zones can correctly summarise the epidemiological characteristics within a certain region.

Climate zones in our study exhibited similar primary peak times but differed in secondary peaks and duration. Different from the peak of influenza activity in the northern provinces/autonomous regions/municipalities in winter and the spring in the southern reported in previous studies, our research shows that different meteorological regions in the north and south have activity peaks in winter and spring, and the peak activity levels in winter are higher than the peak level in spring. Our findings suggest that far northern regions with dry and cold winters earlier showed the sign of influenza epidemic transmission and reached the peak than central and southern regions in Chinese Mainland. Such results fit the previous conclusion in laboratory experiments that dry and cold conditions are favoured by influenza transmission [26–28].

Notably, all climate zones exhibited a significant increasing trend in the 2017–2018, which was observed in both northern and southern regions. Multiple literatures also reported such changes [29–31]. The rapid growth trend since the start of 2019 may be attributed to the widespread application of fast testing methods and the expansion of cover area of sentinel hospitals or surveillance spots [2, 32].

Spatial clustering analysis revealed distinct high-risk clusters in different climate zones. Another study which also utilized the Köppen Geiger system across eight climate zones in Gansu Province (mid-northwest China), arrived at similar results that Dwb zones is the most important area in influenza transmission as it had highest temporal indices and average incidence rate in this province [7]. Both Dwb and Dwc are snow climate with dry winter but slightly differ on summer average temperature (cool or warm summer).

In a nationwide spatiotemporal analysis of influenza, high-risk areas clustered in the central part of China and the lowest-risk areas in the northeast and west [33]. The high risk cluster all located in middle part of China, where the geographic line splitting China to the north and the south crossed [34]. Such differences between south and north had been widely reported [35]. Multiple studies believe southern China had less transmissibility but more frequent epidemic where surveillance should be strengthened [20, 33]. Besides, the latest nationwide surveillance report on Influenza type A reported the high-high risk cluster in 2016–2017 is in Dwa and showing the trend of moving to southern regions [31, 36].

In contrast to previous influenza early warning or forecasting studies primarily conducted at the administrative area level, our research highlighted the significance of climate zones in shaping the seasonality of influenza transmission [37, 38]. A similar study that employed latitude-based climate zone classification illustrated the spatial pattern of influenza-like illness (ILI) cases in Chinese Mainland, indicating that the transmission features in the northern mid-temperate region and the southern subtropical area covered a large part of Chinese Mainland, had the very similar conclusion on our spatial features conclusions [39]. Our study demonstrated the feasibility of early warning for seasonal influenza using the time series characteristics of different ecological-based climate zones and it is a better method to describe the spatial variation of seasonal influenza clusters.

An epidemiological nationwide study on seasonal influenza in China provided detailed spatiotemporal distribution maps from 2014 to 2018 [6]. From this study, we noticed some different provinces/autonomous regions/municipalities shared very similar epidemiological features that were not summarized as one cluster before [6]. The arid climatic regions of northwest China (BWk, BSk) and the continental climatic regions of northeast China (Dwa, Dwb and Dwc) have earlier epidemic waves than the southern regions, and both have only a single winter-spring epidemic season, as described by Zhu et al., in their study of the epidemiological characteristics of northern China. Temperate climate (Cwa, Cwb and Cfa) areas, corresponding to southern China, usually had a high incidence rate and transmissibility of seasonal influenza. Tropical climate area (Aw) had unique epidemiological temporal characteristics as more epidemic peak numbers and circulating durations.

We further reported the spatial distribution variation of age groups in climate zones, which were rarely discussed. The results showed comparing to other age groups, the 0–14 years old age group had a higher risk of influenza infection, especially in areas with warm and wet climates. The much higher incidence rate in the 0–14 age groups indicating the young age groups remain the most vulnerable population group when facing influenza epidemic infection risk. Age group above 65 years old had a higher risk who lived in northern Chinese Mainland (Dwa, Dwb, BSk). A study in Shenyang, a city in northeast China located in Dwa, showed young children had higher ILI percentages in summer and autumn while the elderly usually had high ILI notifications in spring and winter [30].

Furthermore, our study simultaneously explains some conflicting conclusions from previous studies. Previous studies conducted in Shanghai (SH, eastern China), Hong Kong (Hong Kong, southern China) and Panzhihua (PZH, southern west China) were all classified as subtropical zones but corresponding to the Cfa (SH) and Cwb (HK, PZH) zones, respectively in our study, reported different effects of relative humidity on enhancing influenza transmission [4, 27]. This discrepancy may be attributed to the differences in full-year humid air (Cfa) and dry winter (Cwb) between the two zones.

This study is one of the first systematically summarized the spatiotemporal features of seasonal influenza at county level and pointed out the epidemiological features had potential homogeneity with regional ecological conditions distribution in China. We chose Chinese Mainland as our study site because of its vast size, spanning 11 of the 31 climate zones and including all five major climate types in the Köppen Geiger climate system, which based on the newest ecological data in recent years. The large population also provides a rich demographic sample and ensures a diverse sample included in our study. At the same time Chinese Mainland has complex and diverse socio-environmental conditions that contain multiple potential factors influencing the spread of influenza.

However, this study still has several limitations. First, it did not consider the subtypes of influenza. Some studies have shown that Influenza A had a dual peak epidemic in winter-spring in north and summer seasons in south China and Influenza B is more likely to have a single peak circulation within cold months across whole Chinese Mainland [4, 20, 29, 40]. Yet, the details data were unavailable in the research. Am zone, with only one county and no available county-level age group case data, was excluded from our discussion as its conclusions were deemed non-representative of this climate zone. Second, spatial distribution variations of social and economic factors may also be key influences on transmission. The research provided scientific evidence and framework for developing seasonal influenza early warning system (EWS) based on climate zones. We will incorporate more detailed socio-environmental factors to develop the climate-driven EWS with spatiotemporal models. The approach could play a key role in understanding socio-economical determinants, on seasonal influenza transmission prediction [41]. In future research, potential risk factors such as human migrations, population density and socioeconomic indices, etc., will be included in the platform and further improve the predictive accuracy of model. Based on the current research, an integrated EWS that integrates big data, including internet search queries, mobile data, socio-ecological factors, weather changes and other environmental factors will be the goal of our future research [41, 42].

## Conclusions

This study shows that seasonal influenza has different characteristics between different climatic zones under certain regional divisions of ecological conditions and that these differences in spatial and temporal characteristics may be due to differences in environmental factors within the climatic zones. Such variabilities among climate zones may allow us to use known influenza activities in one or more of these climatic zones or priority surveillance areas, in combination with predictable local meteorological conditions, to make early warning and predictions of influenza peaks arrival windows in other climate zones and to implement public health responses more efficiently.

### Supplementary Information

**Additional file 1: Table S1**. Incidence rate in age groups in each climate zones, 2015–2019 (per 100,000 people). **Figure S1**. Age groups incidence rate spatial distributions (a) 0–14 years old (b) 15–64 years old (c) above 65 years old. **Figure S2**. The seasonal factor of incidence rate by age groups in climate zones, 2015–2019 (a) 0–14 years old (b) 15–64 years old (c) above 65 years old. **Figure S3**. The cross-correlation analysis on seasonal factor between BWk and other climate zones.

## Data Availability

The datasets generated and/or analysed during the current study are not publicly available due to the authors do not have permission to share data.

## Abbreviations

IR: Incidence rate
NIDRIS: Notifiable Infectious Diseases Reporting Information System
LISA: Local indicators of spatial association
Am: Tropical, monsoon
Aw: Tropical, savannah
BWk: Arid, desert, cold
BSk: Arid, steppe, cold
Cwa: Temperate, dry winter, hot summer
Cwb: Temperate, dry winter, warm summer
Cfa: Temperate, no dry season, hot summer
Dwa: Continental, dry winter, hot summer
Dwb: Continental, dry winter, warm summer
Dwc: Continental, dry winter, cold summer
ET: Polar, tundra
CW: Cumulative number of weeks in which one or more cases were reported
TW: Total number of weeks over the study period
PV: Total number of prevalent waves
