# Supplementary material for: Epidemiological features of seasonal influenza transmission among 11 climate zones in Chinese Mainland
Source: Infect Dis Poverty. 2024 Jan 10;13:4. doi: 10.1186/s40249-024-01173-9 (PMC10777546; doi:10.1186/s40249-024-01173-9)
Supplement: Supplementary file 1 — Additional file 1: Table S1. Incidence rate in age groups in each climate zones, 2015–2019 (per 100,000 people). Figure S1. Age groups incidence rate spatial distributions (a) 0–14 years old (b) 15–64 years old (c) above 65 years old. Figure S2. The seasonal factor of incidence rate by age groups in climate zones, 2015–2019 (a) 0–14 years old (b) 15–64 years old (c) above 65 years old. Figure S3. The cross-correlation analysis on seasonal factor between BWk and other climate zones. [file 40249_2024_1173_MOESM1_ESM.docx]

Title: Epidemiological features of seasonal influenza transmission among 11 climate zones in Chinese mainland

Authors: Xiaohan Si ^1^, Liping Wang ^2^, Kerrie Mengersen ^3^, Wenbiao Hu ^1^*

* Corresponding author:

Prof. Wenbiao Hu

Ecosystem Change and Population Health Research Group, School of Public Health and Social Work, Queensland University of Technology, Brisbane, Queensland, Australia.

Email: [w2.hu@qut.edu.au](mailto:w2.hu@qut.edu.au)

Author affiliations:

1. Ecosystem Change and Population Health Research Group, School of Public Health and Social Work, Queensland University of Technology, Brisbane, QLD 4059, Australia.
2. Information Center, Chinese Center for Disease Control and Prevention, 102206，Beijing, China.
3. School of Mathematical Sciences, Queensland University of Technology, Brisbane, QLD 4000, Australia.

Table S1. Incidence rate in age groups in each climate zones, 2015–2019 (per 100,000 people)

|  | Maximum incidence rate | | |  | Average incidence rate | | |
| --- | --- | --- | --- | --- | --- | --- | --- |
| Climate zones | 0–14 | 15–64 | 65– |  | 0–14 | 15–64 | 65– |
| Aw | 12.12 | 0.51 | 0.59 |  | 5.64 | 0.33 | 0.38 |
| BWk | 7.76 | 0.72 | 0.91 |  | 1.87 | 0.20 | 0.30 |
| BSk | 24.51 | 2.47 | 3.70 |  | 2.55 | 0.45 | 0.77 |
| Cwa | 60.57 | 6.18 | 5.05 |  | 6.21 | 0.56 | 0.63 |
| Cwb | 1.93 | 0.22 | 0.62 |  | 0.86 | 0.10 | 0.12 |
| Cfa | 41.84 | 2.70 | 2.94 |  | 6.23 | 0.49 | 0.56 |
| Dwa | 41.27 | 3.88 | 4.04 |  | 2.58 | 0.27 | 0.38 |
| Dwb | 3.73 | 0.77 | 1.26 |  | 1.09 | 0.24 | 0.34 |
| Dwc | 3.46 | 0.93 | 0.89 |  | 1.28 | 0.40 | 0.37 |
| ET | 5.53 | 0.34 | 0.21 |  | 0.99 | 0.11 | 0.07 |

Abbreviation list

Am: Tropical, monsoon

Aw: Tropical, savannah

BWk: Arid, desert, cold

BSk: Arid, steppe, cold

Cwa: Temperate, dry winter, hot summer

Cwb: Temperate, dry winter, warm summer

Cfa: Temperate, no dry season, hot summer

Dwa: Continental, dry winter, hot summer

Dwb: Continental, dry winter, warm summer

Dwc: Continental, dry winter, cold summer

ET: Polar, tundra


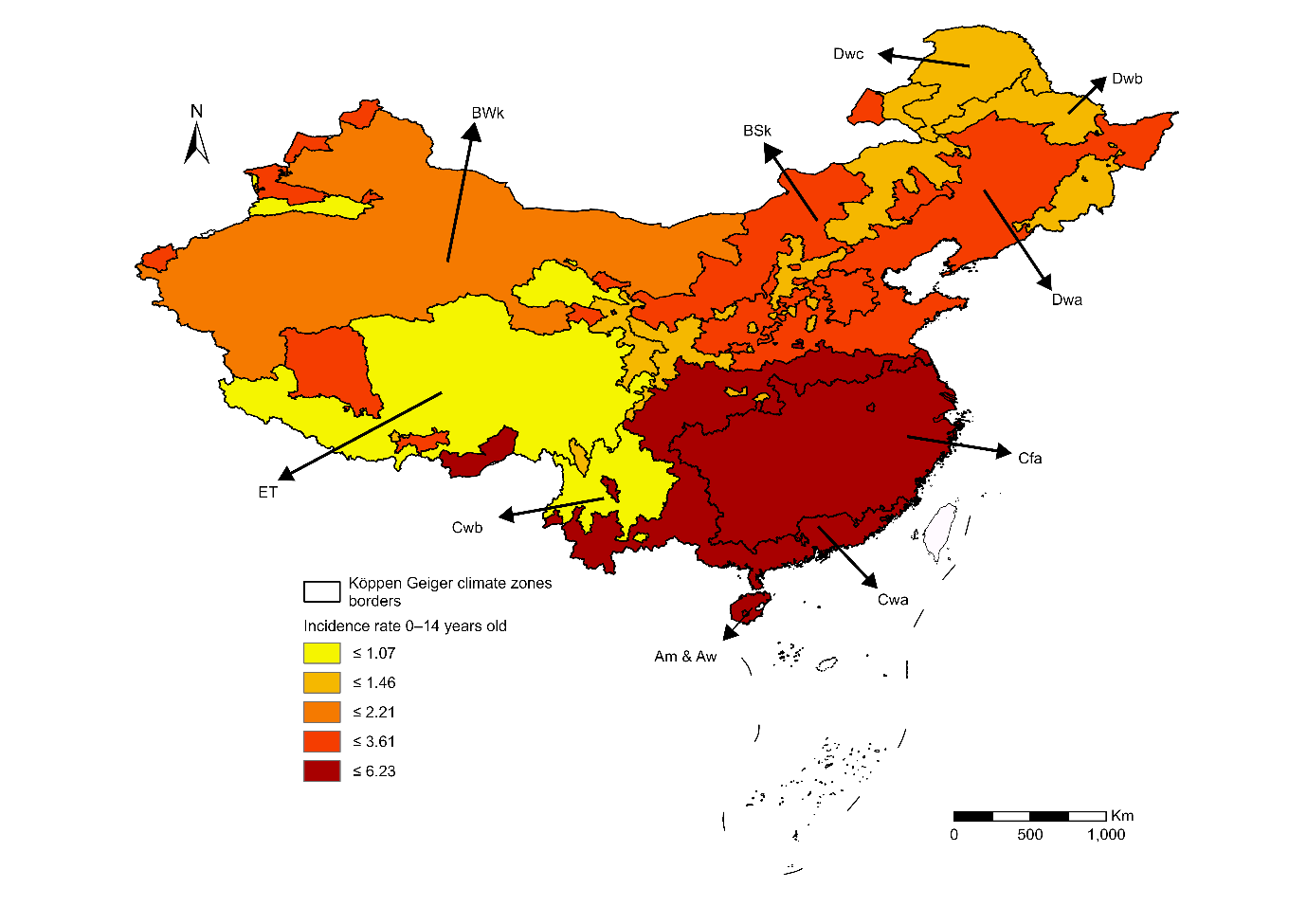


(a)


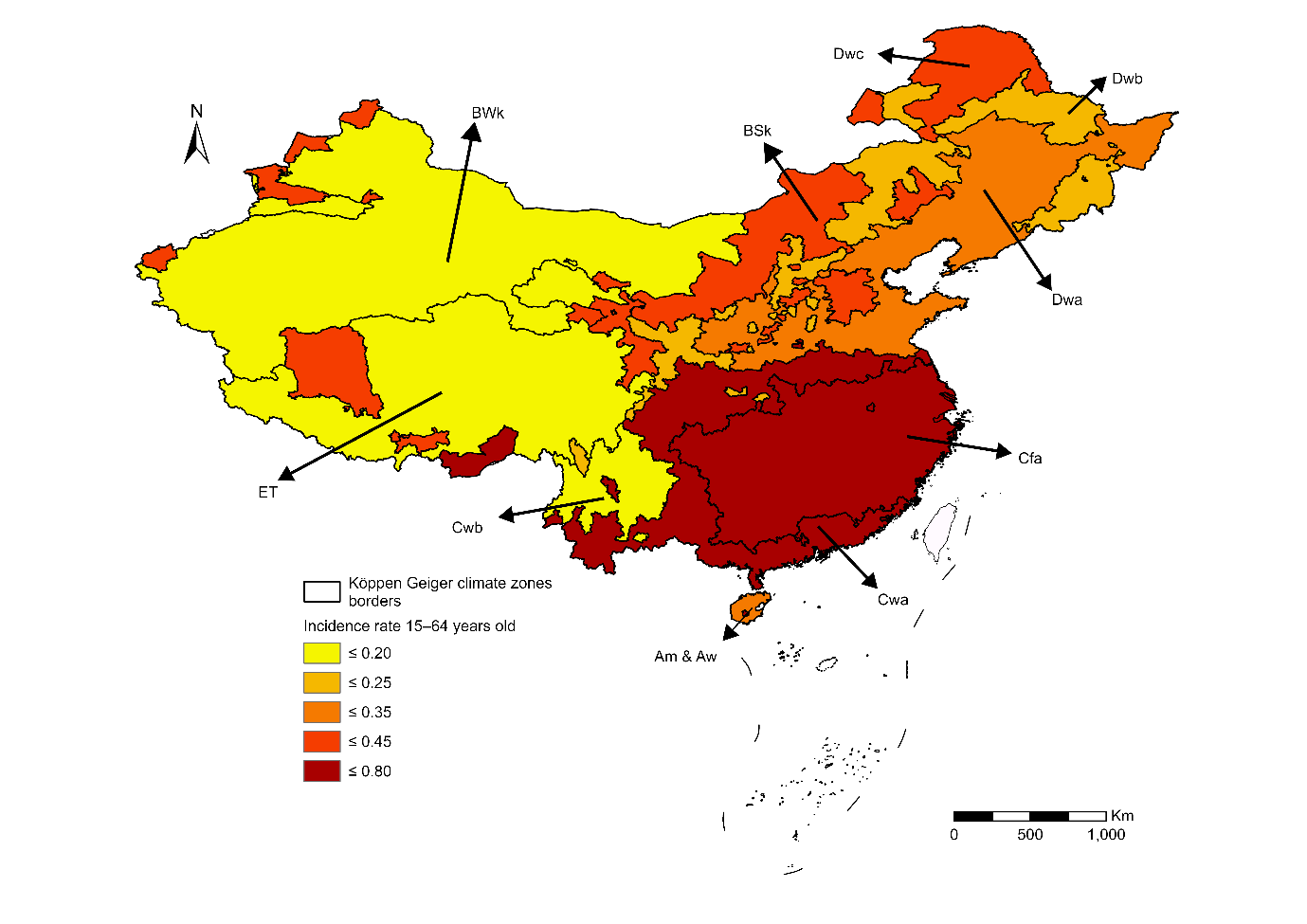


(b)


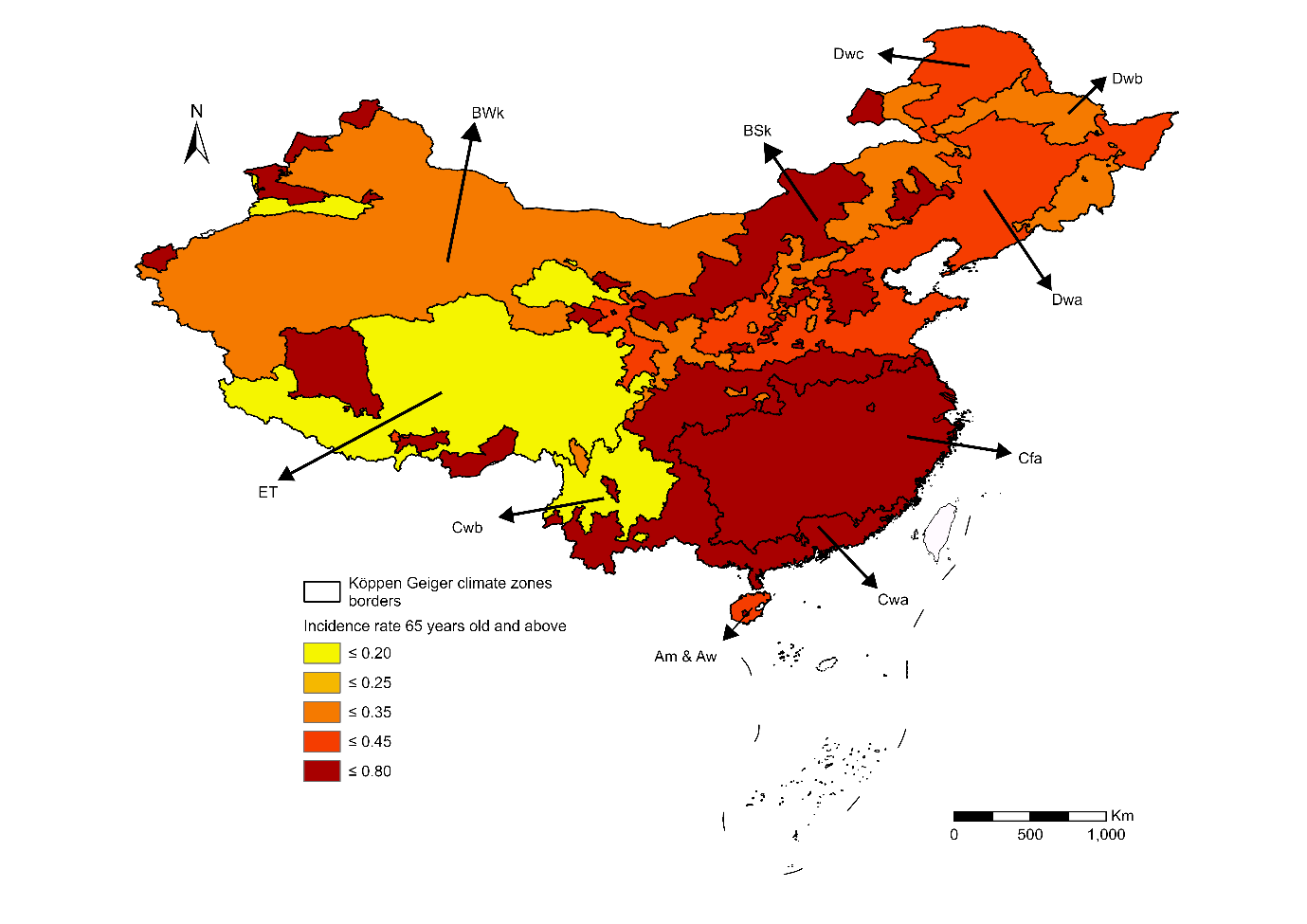


(c)

Figure S1. Age groups incidence rate spatial distributions (a) 0–14 years old (b) 15–64 years old (c) above 65 years old.


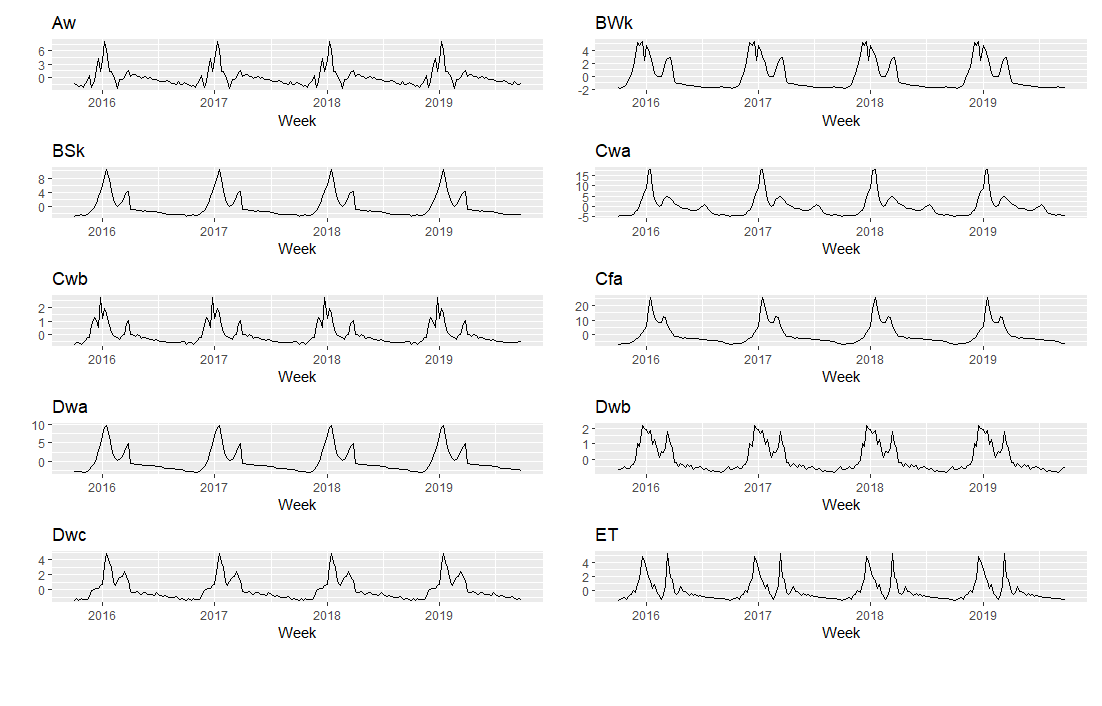


(a)


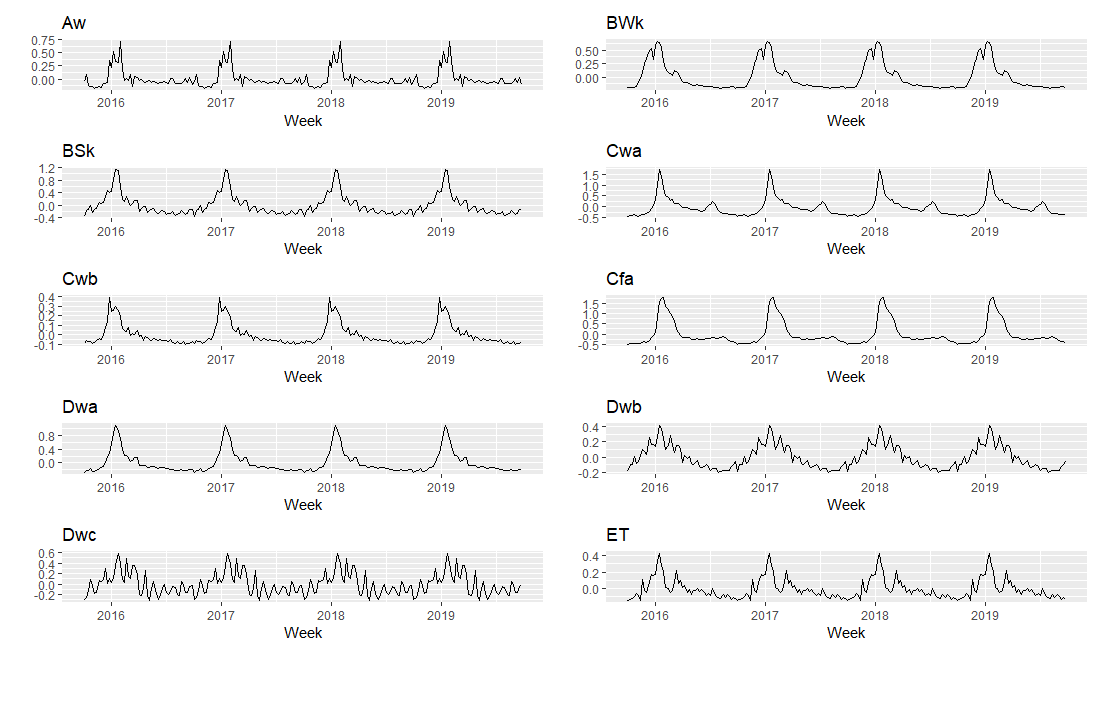


(b)


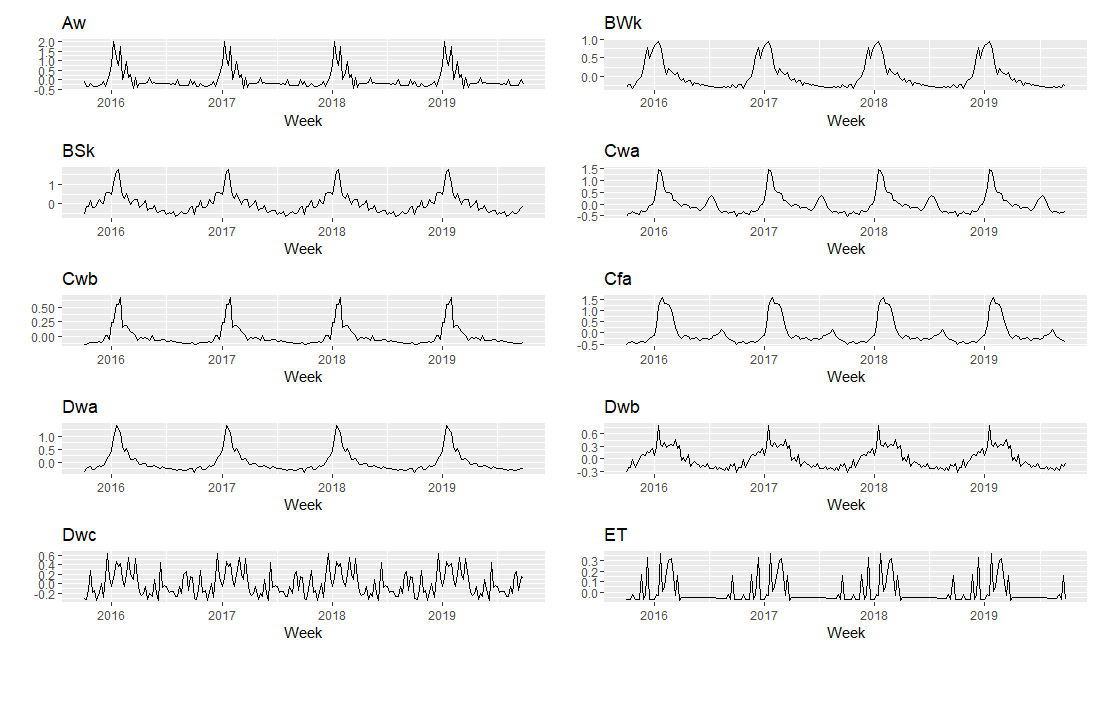


(c)

Figure S2. The seasonal factor of incidence rate by age groups in climate zones, 2015–2019 (a) 0–14 years old (b) 15–64 years old (c) above 65 years old


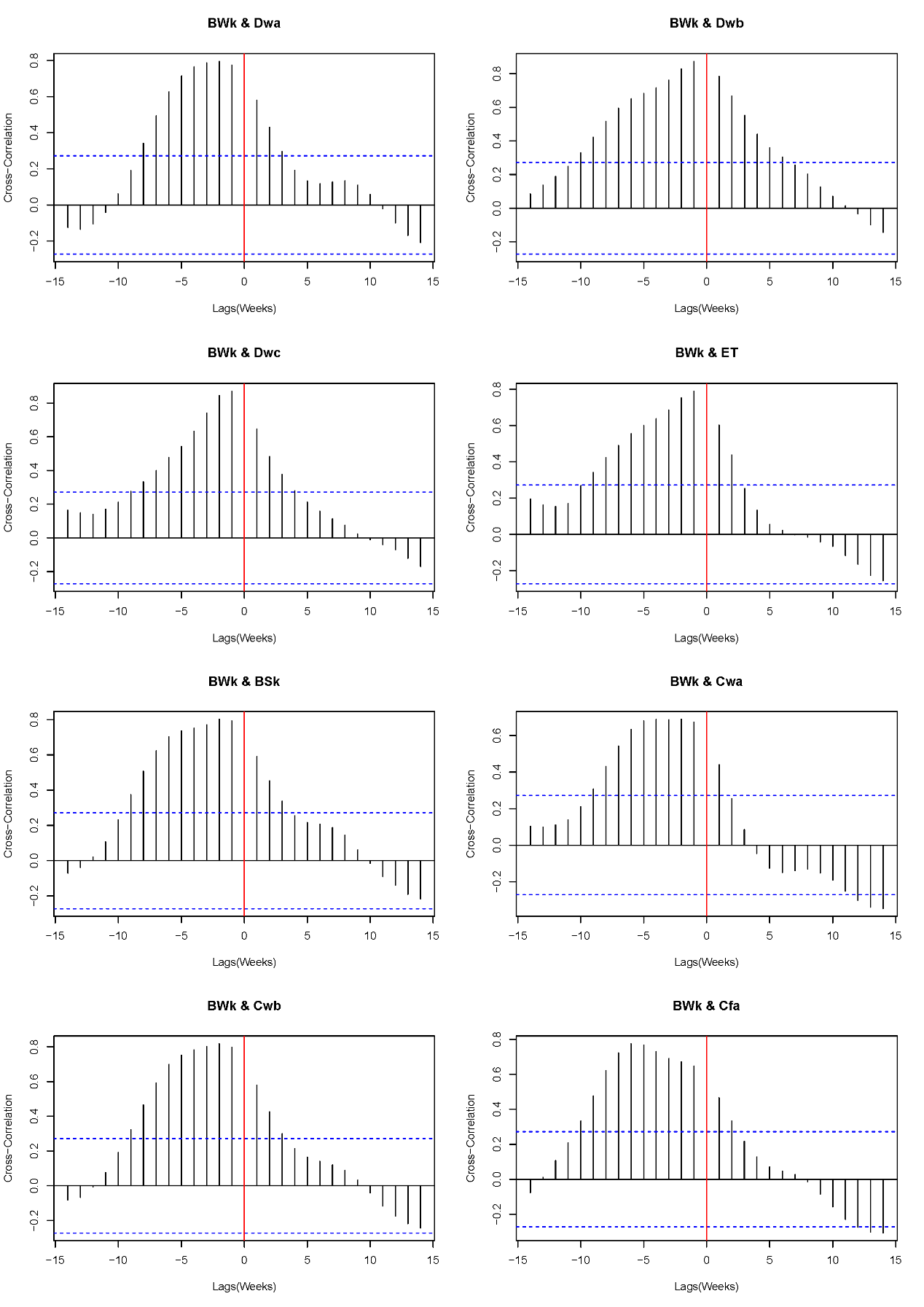


Figure S3. The cross-correlation analysis on seasonal factor between BWk and other climate zones.
